# Protein, amino acids and obesity treatment

**DOI:** 10.1007/s11154-020-09574-5

**Published:** 2020-08-21

**Authors:** Mathilde Simonson, Yves Boirie, Christelle Guillet

**Affiliations:** grid.494717.8 0000000115480420 UNH, Unité de Nutrition Humaine, CHU Clermont-Ferrand, Service de Nutrition Clinique, CRNH Auvergne, INRA, Université Clermont Auvergne, 63000 Clermont-Ferrand, France

**Keywords:** Amino acids, Proteins quality and quantity, Obesity, BCAA, Methionine, Tryptophan

## Abstract

Dietary proteins have been used for years to treat obesity. Body weight loss is beneficial when it concerns fat mass, but loss of fat free mass – especially muscle might be detrimental. This occurs because protein breakdown predominates over synthesis, thus administering anabolic dietary compounds like proteins might counter fat free mass loss while allowing for fat mass loss.

Indeed, varying the quantity of proteins will decrease muscle anabolic response and increase hyperphagia in rodents fed a low protein diet; but it will favor lean mass maintenance and promote satiety, in certain age groups of humans fed a high protein diet. Beyond protein quantity, protein source is an important metabolic regulator: whey protein and plant based diets exercize favorable effects on the risk of developing obesity, body composition, metabolic parameters or fat free mass preservation of obese patients. Specific amino-acids like branched chain amino acids (BCAA), methionine, tryptophan and its metabolites, and glutamate can also positively influence parameters and complications of obesity especially in rodent models, with less studies translating this in humans.

Tuning the quality and quantity of proteins or even specific amino-acids can thus be seen as a potential therapeutic intervention on the body composition, metabolic syndrome parameters and appetite regulation of obese patients. Since these effects vary across age groups and much of the data comes from murine models, long-term prospective studies modulating proteins and amino acids in the human diet are needed.

## Introduction

Obesity has become a major public health problem because of its high prevalence in both developed and developing countries and its major complications, such as diabetes mellitus, cardiovascular diseases, respiratory failure and cancers. Finding effective treatment and/or preventive approaches targeting weight loss, its maintenance, and thus the reduction of obesity prevalence is a major challenge.

Obesity is accompanied by important changes in body composition reflecting a combination of changes in fat mass (FM) and fat free mass (FFM), leading thus to modification of energy expenditure and regulation of food intake and appetite. Obesity is also associated with various degrees of metabolic impairments especially regarding carbohydrate and lipid metabolism, but it is noteworthy that protein metabolism is also affected in this clinical situation. Indeed, protein turnover rate involving protein synthesis and protein breakdown processes, modulates muscle quantity and quality by controlling the amount of proteins and their post-translational modifications in skeletal muscle. On a day-to-day basis, hormones, nutrients and physical exercise tightly regulate protein metabolism. After meal intake, amino acids coming from dietary proteins digestion together with the secretion of insulin are strongly involved in the regulation of protein metabolism. Changes in protein metabolism influence whole body nitrogen balance and contribute to modification of protein nutritional requirements for a specific population. Alterations in the regulation of protein metabolism have been demonstrated in obese subjects, resulting in a lesser inhibition of proteolysis and a normal or lower stimulation by insulin and amino acids in the whole body [1]. At skeletal muscle level, obesity is associated with a reduction in the rate of protein synthesis in the fasted state and to its response to increased plasma amino acid concentrations [2] and to nutrition and exercise [3]. These variations in protein metabolism observed in obesity might be explained not only by the metabolic disorders that accompany obesity, like insulin-resistance or inflammation, but also by body composition changes occurring during weight fluctuation. Considering these changes for body composition, energy expenditure, regulation of protein intake and appetite, and regulation of protein metabolism, it is thus of major importance to consider specific dietary approaches based on protein and amino acid intakes to achieve weight loss or weight maintenance for patients with obesity.

## Using proteins to treat obesity

### Dietary protein quantity and obesity

#### Potential role of low protein intake

An interesting hypothesis suggests that unbalancing the protein/non protein ratio intake in a diet is compensated by overfeeding and increased energy intake, and may play a key role in the development of obesity [4, 5]. This theory emerged several years ago and is known as the protein leverage hypothesis. In fact, a decrease in the proportion of protein within a diet drives excess energy intake with non-protein energy nutrients (carbohydrates and fats) to compensate the energy deficit from reduced protein intake [6]. This phenomenon is not observed with carbohydrates or fats. In humans, using the QUALITY Canadian prospective study, Roberge et al. reported that a lower consumption of dietary protein in 8–10 years old youths tends to be associated with increased risk of developing metabolically unhealthy obese phenotype at age 10–12 [7]. A cross-sectional study from the Korean National Health and Nutrition Examination Survey data studied dietary macronutriment intakes and phenotypes of obesity. Low protein intake was positively associated with metabolically healthy obesity in women [8]. In rats, mild (10% of total energy intake (TEI)) to moderate protein restriction (5% of TEI) is associated with hyperphagia and increases energy expenditure through activation of the sympathetic system [9]. Additionally, authors found similar lean mass in the group of rats with a 10% protein diet matched in protein intake to the 5% protein group, suggesting that the intake of dietary protein, but not calorie intake is necessary to preserve lean body mass. So protein restriction per se seems to be responsible for disturbing calorie intake and energy expenditure in animals. In humans, the relation between low protein intake and appearance of metabolic disorders in obese individuals, and the implication of reduced protein intake on increased risk of obesity is not yet clear. Thus, we need more prospective studies to understand how protein intake influences the development of obesity phenotypes.

#### High protein intake

Considering the properties of dietary protein in promoting satiety, energy expenditure through the regulation of meal-induced thermogenesis and in changing body composition in favor of lean mass, high protein diets might be interesting for the management of obesity [10]. In obese adolescents, high protein increased meal-induced thermogenesis and fullness scores [11], whereas no effect on regulating appetite hormones (GLP1, PYY) and energy intake were observed with the high protein diet [12]. Another study reduced calories for a year to 1200–1800 kcal/day, while keeping the normal protein intake for 21 severely obese children and adolescents [13]. Diet adherence was limited, but patients lost an average of 4.7 kg and family quality of life improved.

This beneficial effect might be true for adolescent but not for younger children. In the European CHOP study, a double blind, randomized prospective clinical trial enrolling healthy newborns fed higher or lower protein content formula versus breast-feeding, children’s plasma metabolome at 5.5 and 8 years old is similar [14]. Additionally, comparing HP with LP formula or breast-feeding during infancy yields an OR of 2.87 and 2.84 for developing obesity at 6 years of age, respectively. During the perinatal period, the exposure of foetus to low or high protein diet might influence metabolic phenotypes, predisposing to metabolic disorders in adulthood. Indeed, in a murine study, authors gave a 55% protein diet or a normal protein diet during gestation, a normal diet during weaning, and a normal protein diet or macronutrient choices after weaning [15]. They found high protein diet during gestation was associated with higher body weight and visceral adiposity and altered insulin liver signaling in pups given the choice between the three macronutriments. Therefore, the beneficial effect of high protein diet interventions in adolescence could be deleterious during infancy and pregnancy: however, studies still need to confirm the exact age where the  effect changes.

In older obese adults, increasing protein intake is needed for conserving lean body mass, especially in case of weight loss or when undergoing caloric restriction. In a 6 months randomized controlled trial, 96 men and women with class I or II obesity aged over 65 years old underwent or not a dietary weight loss intervention with 1100 to 1300 kcal per day and a high protein amount (1.2–1.5 g/kg body weight/day). Mean weight loss was 1.16 kg over 6 months, 87% of it being fat mass loss according to DEXA measurements. Lean body mass was preserved and mobility slightly increased during weight loss [16]. Moreover, the android to gynoid fat mass ratio improved, in association with subsequent lipid and glucose plasmatic profiles [17]. Finally, the hypocaloric, nutritionally complete, high protein diet helped to maintain bone density and quality during weight loss [18]. In a recent meta-analysis, Hsu et al. also found that very low calorie, high protein diets improve body composition compared to very low calorie, normal protein diets, preserving skeletal muscle mass and decreasing fat mass [19]. These results for diet interventions using high protein intakes in older obese patients are encouraging, enabling the beneficial loss of fat without the deleterious loss of lean mass and the preservation of muscle and bone quality during weight loss.

### Protein quality impact during obesity

#### Whey protein

Beside the quantity of protein consumed, the quality of protein - i.e., its digestibility and its composition in essential amino acids - might have differential effects on metabolism or body phenotype in obesity. With casein, whey protein is one of the two main proteins in milk, which possesses the highest satiating properties compared to other protein sources [20, 21]. Whey protein has been characterized as a “fast” digestible protein enriched in leucine, which activates post-prandial protein synthesis and presents beneficial effects on the preservation of lean body mass in older people [22, 23]. Urinary metabolic analyzes on Sprague Dawley rats fed a HFD with 15% protein consisting of whey or beef showed lower carnitine, tyrosine and phenylalanine metabolites and creatine/creatinine excretion as well as higher tryptophan metabolites: so depending on the protein source, the effect on amino-acid metabolism may vary [24]. A recent meta-analysis of 37 randomized controlled trials pooling 2344 individuals reviewed how supplementing whey protein for overweight and obese individuals affected their metabolic syndrome. In this population, whey protein supplementation improved waist circumference, blood pressure, and lipid or sugar plasmatic levels, despite heterogeneity in protein doses, BMI, age and follow up duration [25]. Additional studies on body composition showed fat free mass stayed similar for obese adults when adding whey protein supplementation to a four-week long, very low calorie diet and exercise intervention, albeit the statistical power for this study was insufficient with approximately 15 participants per group [26]. In another study by Giglio and colleagues, 52 obese women were randomized and supplemented with either whey protein or hydrolyzed collagen as an afternoon snack for 8 weeks [27]. Fat free mass did not differ between groups and android fat measured by DEXA decreased in the group supplemented by whey.

Additional proof of whey protein’s benefits on lipid levels was brought by a randomized controlled trial by Rakvaag et al. [28]. Obese and overweight adult patients added whey protein or maltodextrin and varying amounts of fiber to their diet. Fasting and postprandial lipid profiles in plasma improved in the group with whey protein and low fiber [28].

Recent studies also focused on whey protein impact on specific age groups. In 9 adolescent obese women, enriching a drink with whey protein - used as preload for lunch - incurs less hunger with more satiety sensations, lower blood sugar levels and higher anorexigenic hormone secretion compared to a maltodextrin-enriched beverage [29]. In older sarcopenic populations, a meta-analysis focused on the effect of exercise combined with nutrition in sarcopenic obese subjects [19]. They found that protein supplementation combined with exercise did not add any positive effects concerning body composition, muscle function, metabolic or inflammatory biomarkers. However, this data was extracted from 153 patients in three randomized controlled trials [8, 30, 31] and the study with the highest weight used essential amino-acids [8]. Maltais et al. however randomized 26, overweight, sarcopenic men who performed resistance training for 16 weeks, delivering a post-exercise dairy, non-dairy shake or rice milk as a control shake [31]. Resistance training significantly increased lean mass measured by DEXA in all three groups, but patients fed the dairy shake also significantly decreased their fat mass, with an increase in muscle mass to fat mass ratio in this group. Another randomized clinical trial included 26 sarcopenic obese women to receive placebo or hydrolyzed whey protein, with 12 weeks of resistance training, and revealed a greater, significant increase of appendicular lean soft tissue - composed by skeletal muscle, skin, connective tissue and tendons - and total, trunk fat mass and consequently sarcopenia frequency decreased in the whey group compared to placebo [30].

To summarize, the main effect of supplementing with whey protein in obese humans is a decreased body fat mass and improvement of metabolic syndrome parameters. Coupled with calorie restriction and exercise these favorable effects persist and additionally, fat free mass is maintained. In specific age groups, whey protein benefits satiety and sugar levels in adolescents, and improves body composition while decreasing sarcopenia when combined with resistance training in older patients.

#### Plant-derived protein

Using animals as protein sources burdens the environment, whereas plants are also interesting protein-sources and more environment-friendly [32]. Plants have lower essential amino-acid content such as leucine, isoleucine and valine, lysine and methionine compared to animal sources of protein and are less digestible compared to animals, so the final amount of amino-acid extracted for anabolic use is lower [33]. However, combining legumes and cereals for instance can counter these limitations and avoid amino-acid imbalance [34], and plant-based diets actually reduce body weight and body fat, which is interesting to treat obesity [35]. In favor of whether plant-based diets benefit obese patients, Navas-Carretero and colleagues exploited the Food4Me study [36]. This study included 2413 European adult participants who completed online surveys of morphological data, dietary intake, and medical comorbidities at multiple time points during a 6 month intervention of personalized nutrition [37]. Comparing the population using a 25 kg/m^2^ BMI cut-off, authors revealed subjects with the highest BMI had the highest animal protein consumption and the less vegetable protein one; moreover, substituting 1% of animal protein by vegetable protein reduced the risk of becoming overweight or obese.

Looking at interventional studies, an 8-weeks whole-food plant based diet group intervention in 78 participants of varying BMI, who were vegan, vegetarian or non-vegetarian at baseline induced a two-point BMI loss and improved lipid plasmatic profiles as well as mean blood pressure parameters, with a lesser effect when patients were already vegan or vegetarian at baseline [38]. In another 16 weeks randomized controlled trial, only obese and overweight adults were fed a vegan diet or their classical diet, and in the vegan group patients lost 2 points of BMI, 4.3 kg of DEXA-measured fat mass and reduced their insulin resistance index compared to the control group [39]. Of note, fat mass decrease correlated with the decreased intake of animal protein as well as leucine and increased intake of plant protein, and specific amino acids like leucine correlated with fat mass increase, while histidine correlated positively with insulin resistance decrease. Mechanistically, an explanation might come from differences in gastrointestinal hormone secretion and satiety sensations depending on the protein’s source. In a 60 male adult patient intervention, with a type II diabetes group, a BMI and age-matched group and an age-matched, healthy group, giving two test burgers containing tofu or meat and cheese at a one-week interval, in random order, led to higher satiety feeling, post prandial GLP1 and amylin levels in the group that took tofu [40]. Since GLP1 and amylin are hormones involved in the pathway inducing satiety, this might partially explain the effect of vegetable proteins on body weight. Concerning NASH and plant protein sources, Deibert and his team compared lifestyle changes and yogurt enriched with soy as meal replacement in 22 obese patients for 24 weeks [41]. Replacing meals by soy protein and encouraging lifestyle changes led to equivalent, beneficial effects on body weight, intra-liver lipid content, and subcutaneous fat mass compared to baseline. Soy protein had a stronger effect on reducing waist circumference and triglyceride levels compared to the latter group.

Summarizing, evidence suggests vegetable protein sources benefit the body composition, metabolic parameters and liver steatosis of obese patients. Studies with longer interventions coupled with multiple gastro-intestinal hormone and satiety measures should be encouraged before putting this therapeutic intervention to use.

### Bariatric surgery and protein changes

Since protein quality and quantity are key for the health of obese patients, how do therapeutical interventions for obesity like bariatric surgery impact proteins' digestion and metabolism? As reviewed recently, Roux-en-Y gastric bypass (RYGB), the gold standard surgery, has restrictive and malabsorptive consequences: concerning proteins, the gastric pouch becomes smaller so protein intake decreases, gastric enzymes necessary to activate pancreatic protein digestion enzymes might be less secreted with a potential delay in RYGB between protein ingestion and their interaction with pancreatic enzymes [42]. Sleeve gastrectomy, more widely practiced potentially because it is only restrictive and not malabsorptive, might have the same impact on protein ingestion and pancreatic enzymes [42]. Taking these changes into account a minimum of 60 g of protein per day becomes optimal, the adequate intake going up to 1.5 g of protein/kg of ideal body weight per day [42]. A recent study has determined protein requirement in morbidly obese patients before, 3 and 12 months after surgery using a validated method such as nitrogen balance. The calculated need values indicate higher than expected protein requirements both at 3 and 12 months. This explains the need to further strengthen the post-bariatric protein intake and allows new recommendations to be made [43]. Postoperatively, lower protein intake leads to decrease in fat free mass, increasing the risk for sarcopenic obesity [44]. Higher intakes of dietary protein or protein supplementation following bariatric surgery are associated with a higher, long term weight loss and fat mass loss [45–47]. Two recent prospective studies following obese patients after RYGB or sleeve gastrectomy corroborated that high protein intake was associated with higher total weight loss, at 3 and 18 months post-operatively [48, 49]. A cross-sectional study in 60 women without an operation versus women 2 years after RYGB, who regained weight or not, showed lower protein intakes increased the risk of weight regain and were correlated with lower satiety sensations after a test meal [50]*.* Interestingly, in a retrospective study of 100 patients after RYGB or sleeve gastrectomy, Smelt and colleagues found that post-operative handgrip strength, reflecting muscle strength, was impacted significantly by protein intake [51]. Oppert and colleagues went further with an 18-week, randomized, controlled trial assigning 26 obese women operated by RYGB to a control group, a group supplemented with whey protein, two powder drinks per day at a dose of 48 g/day, and a group combining resistance training 3 times per week with whey supplementation [52]. While they did not uncover a significant difference in lean mass loss between groups, for patients completing the whole study the muscle strength in lower and upper limbs relative to body weight and the protein intakes improved significantly in the group combining exercise and protein supplementation.

Mechanistically, changes in food tolerance or hunger/satiety did not explain how protein intake affected the post-operative study comparing RYGB and sleeve gastrectomy at 18-months in 39 patients, or the cross-sectional study aforementioned [49, 50]. Moreover, Janmohammadi meta-analyzed the impact of bariatric surgery type on macronutrient intake: protein intake decreases post operatively in all surgery types, but this decrease did not differ significantly between surgery types [53]. Golzarand and colleagues corroborated this finding in a 6 month prospective study of 43 patients operated by sleeve gastrectomy or RYGB: postoperative protein intake decreased in both groups but protein intake, its proportion of energy out of the total energy and body composition did not change significantly between surgery type groups [54]. Therefore, protein intake is diminished in the initial phases after bariatric surgery but should not decrease too much since this affects the surgical outcome. Food preference changes, nutritional sensations and surgery type do not explain this. Finally, protein supplementation combined with resistance training might increase muscle function and prevent fat free mass loss.

## Using specific amino acids to treat obesity

### Amino acid composition, changes in obesity and after therapeutic interventions

Beyond protein intake, how are individual amino acids important in obesity? It has long been known that plasma amino acid profiles are changed during obesity and are linked with changes in insulin secretion and the development of insulin resistance. A Chinese study by Okekunle and colleagues recruited 9734 patients aged 20 to 74 years old via questionnaires between 2010 and 2012 [55]. Then 4118 respondents: 3009-normal weight and 1109-obese were included. Obesity risk was inversely associated with branched amino acids, lysine, phenylalanine, threonine, histidine, cysteine, tyrosine, proline, serine, and diacid aminoacids. This is interesting since models of DIO in rodents find a high fat diet induces higher liver levels of branched amino-acids, alanine, glutamate and methionine, with decreased levels of glycine and taurine [48]. Other authors focused on obtaining metabolomics AA signatures that differentiate weight regain after RYGB [57]. Controls, 22 patients with weight regain and 14 patients with sustained weight loss were compared several years after the intervention; weight regain was associated with decrease in metabolites related to serine, glycine, threonine pathways, alanine, phenylalanine and finally glutamate metabolism, suggesting the lower these metabolites, the higher the weight regain.

**Fig. 1:**
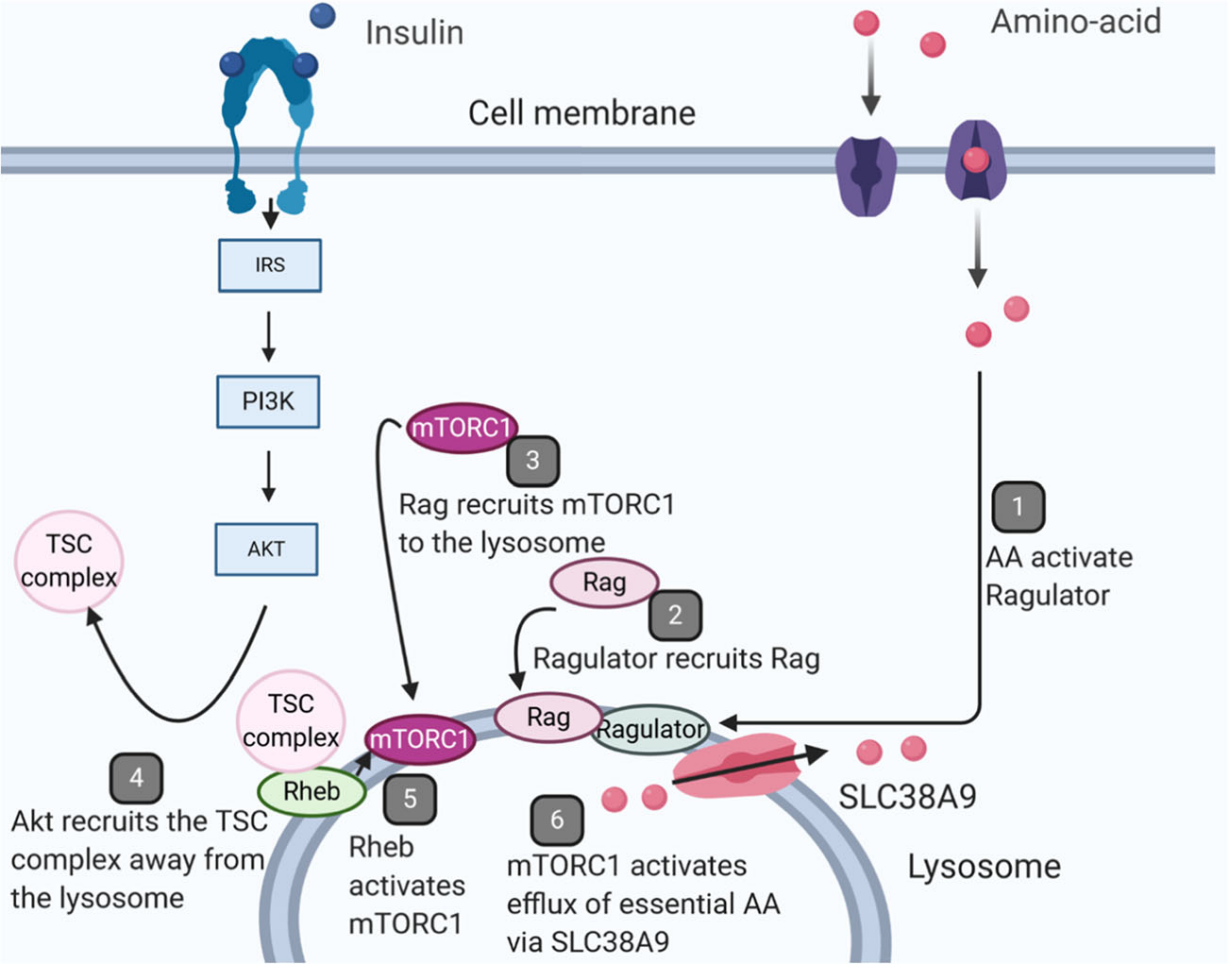
Amino-acid sensing and the mTOR pathway – adapted from Green et al. and Wyant et al. MTORC1: mechanistic target of rapamycin complex one; IRS: insulin receptor substrate protein; PI3K: phosphoinositide 3-kinase; AKT: protein kinase B. *AA* amino-acid, *TSC* tuberous sclerosis complex. Figure constructed using the Biorender software

Mechanistically, amino acids are involved in the insulin signaling pathway by interacting with the target of rapamycin (mTOR) complex. mTOR can form two complexes, I (mTORC1) and II (mTORCII), the former of which integrates amino-acid availability to induce protein synthesis [58, 59]. After insulin binds the insulin receptor, the insulin receptor substrate (IRS) protein is activated, triggerring the phosphoinositide-3-kinase (PI3K)/AKT pathway [60] (Fig. 1). Akt activation leads to moving the TSC complex, inhibitor of RHEB, from the lysosome to the cytoplasm, disinhibiting RHEB to activate mTORC1 [61]. On the other hand, amino acids in the cytosol recruit mTORC1 to the lysosome via a GTPase, Rag, whose lysosomal localization is controlled by the Ragulator complex [61]. Once mTORC1 is located at the level of lysosome, it mediates the efflux of essential amino-acids such as leucine, isoleucine, methionine and tryptophane via arginine activation of SLC38A9, a transmembrane lysosomal transporter: the amino-acids are now available for use [62]. The complex mTORC1 also crosstalks with FGF21, a liver protein secreted during fasting which extends lifespan, improves insulin sensitivity and diminishes adiposity and liver steatosis [63, 64]. In T3-L1 adipocytes, Minard and colleagues mapped the FGF21 signaling network by labeling stable isotopes in cell cultures and stimulating the cells by FGF21 [65]. Analysis of the phosphorylated peptides in these cells using proteomics revealed mTORC1 as the third most phosphorylated protein by FGF21 and the same results were observed in mice injected with FGF21. This action of FGF21 on mTORC1 was mediated by another kinase, Mitogen–activated protein kinase (MAPK). Finally, inhibiting mTORC1 via use of rapamycin in vitro on cultured adipocytes impaired FGF21-induced glucose uptake, adiponectin secretion and uncoupling protein 1 expression, a protein important for adipocyte browning. So mTORC1 is a major protein in amino-acid sensing pathways which has beneficial effects on metabolism through crosstalks with FGF21.

### Branched chain amino acids

Both animal and human studies suggest an important role for branched chain amino acids (BCAA) in the pathogenesis of metabolic disorders observed in obesity and diabetes [66]. The observation of higher circulating BCAA levels in individuals with obesity was first reported by Felig et al. [67]. More recently, an increase in BCAA plasma concentration in older subjects with high body fat and lean mass was reported, suggesting that elevation of BCAA can be also found in older people with obesity but with specific body composition characterized by both an increase in fat and in lean body mass [68]. The increased BCAAs correlated with circulating insulin levels, suggesting the elevation of circulating BCAA concentration is a manifestation of insulin resistance. Metabolomics studies have indicated that increased circulating amounts of BCAAs, Phenylalanine, and Tyrosine were associated with up to a 5-fold increase in risk for future development of Type 2 Diabetes [69] and predicted improved insulin sensitivity in response to a dietary and behavioral weight loss intervention in obese individuals [70]. In the Framingham Heart Study, patients with elevated BCAAs had the highest probability to suffer from diabetes 10 years in the future [71]. Neinast et al. have recently analyzed whole body BCAA metabolism in db/db mice, by using an in vivo isotopes study combined with metabolomics analysis [72]. Db/db mice present an altered distribution of whole-body BCAA oxidation, shifting oxidation away from white adipose and liver toward skeletal muscle. The suppression of oxidation in fat and liver is consistent with the reported reduced expression of specific enzymes involved in BCAA oxidation (BCATs and BCKDH) [73, 74]. Reduced BCAA oxidation in white fat and liver induces BCAA overflow to skeletal muscle, driving its BCAA oxidation at muscle level.

Another potential explanation for elevation of circulating BCAA might involve dysbiosis in gut microbiota. Positive correlations between microbial functions—including BCAA biosynthesis—and IR are largely driven by a few species only, notably *P. copri* and *B. vulgatus*, suggesting that they may directly impact host metabolism. Pedersen et al. [75] have tested this hypothesis in mice fed a high-fat diet, and found that a challenge with *P. copri* led to increased circulating serum levels of BCAAs, insulin resistance and an aggravation of glucose intolerance. They conclude that dysbiosis of the human gut microbiota impacts the serum metabolome and contributes to insulin resistance. However, a recent study performed in overweight and obese adults indicated that the associations of gut microbiota metabolites with diabetes- related outcomes were independent of changes in BCAAs, suggesting other pathways were likely to be involved [76].

In addition, a supplementation of BCAAs in rats fed with a high fat diet contributes to development of obesity-associated insulin resistance [77]. Human observational studies have reported a positive association between dietary BCAA intake and T2DM risk [78, 79]. However, there remain controversies regarding the relation between BCAAs and insulin resistance. Indeed, recent evidences provided that additional BCAA supplementation in obese, prediabetic patients did not impair glucose metabolism [80]. Instead, glucose metabolism tended to be improved after daily additional BCAA supplementation (20 g/day) for 4 weeks. This is likely because BCAA supplementation did not promote an elevation in circulating BCAAs and BCKAs.

### Sulfur based amino acids as a potential therapy for obesity

Beyond restricting protein intake moderately, what happens with severe protein restriction, or total amino-acid restriction? Inducing a three-week, HFD, protein free diet (0% of TEI) in obesity prone CD rats triggered hypophagia, increased energy expenditure, insulin sensitivity, thermogenic and FGF21 gene expression levels in muscle and brown adipose tissue; and decreased body weight, fat and lean mass compared to control rats with 15% of TEI protein intake [81]. Moreover, in these same rats, essential amino-acid levels were lower compared to controls: threonine, tryptophan, valine, phenylalanine, leucine, isoleucine and lysine, histidine and methionine were affected. Since loss of lean mass is involved, one wonders if restricting specific amino-acids like methionine could reproduce the effects of a protein free diet without this deleterious effect. Methionine is an amino acid which is lower in plant protein sources compared to animal protein sources [82]. Its restriction mimics the effects of protein restriction except the food intake increases and lean mass is unchanged [83, 84]. To date, only one randomized controlled double blind trial specific to the obese population allocated 26 adult patients to a 2 mg/kg/day or 35 mg/kg/day methionine diet for 16-weeks, without any significant difference in body weight, insulin sensitivity or fat mass [85].

Thus, for obese patients methionine restriction might become a therapeutic approach since it is more easily attainable than protein restriction, with the goal of improving body composition, metabolic syndrome components, inflammation and oxidative stress [84, 86]. Methionine restriction in rodents reproduces the beneficial effects of total amino acid restriction, on body weight and composition, insulin sensitivity and sympathetically mediated energy expenditure. Compared to total amino acid restriction, methionine restriction can lead to either hyperphagia or transient hypophagia in diet induced obesity rodent models [87, 88]. Methionine restriction improves glucose metabolism and insulin sensitivity in obese mice by a cross talk between the adipose tissue and skeletal muscle, involving an adipose-derived factor, the adiponectin. In vitro, GLUT4-mediated glucose uptake increases and glycolysis is enhanced in skeletal muscle cells incubated with adiponectin [89]. In diet-induced obesity, mice display high levels of plasma adiponectin when restricting methionine, [90, 91]. In male C57BL/6 J mice fed a 10 week long HFD to induce obesity, restricting methionine increases plasmatic but also skeletal muscle adiponectin, downregulates the mTOR pathway and upregulates MAPK and IRS-1 mRNA levels in gastrocnemius muscle, leading to an improvement in insulin sensitivity [92].

In DIO mice models, methionine restriction also improves lipid profiles and hepatosteatosis [56, 88, 90, 93, 94]. Methionine restriction reduced the lipogenesis/lipid catabolism ratio, improving lipid accumulation and thus liver function, in male C57BL/6 J mice fed a 21 week long HFD [56]. It is noteworthy that the methionine and choline deficient diet in mice without DIO is a widely used model to study “lean NASH” [95]. Restricting methionine in C57BL/6j mice fed a 22 week long HFD also impacted liver protein metabolism, since protein turnover improved by increasing retention efficiency compared to controls with obesity but without methionine restriction [85]. Moreover, in this study, liver protein degradation is upregulated and protein synthesis is downregulated, and methionine restriction normalizes these effects.

Concerning inflammation and oxidative stress, DIO leads to higher levels of pro-inflammatory cytokines and lower levels of anti-inflammatory levels and has a negative effect on the pro-oxidant/anti-oxidant stress balance in favor of oxidative stress [88, 94, 96]. Firstly, restricting methionine in C57BL/6 J mice fed a 10 week long HFD lowered pro-inflammatory cytokine levels while raising anti-inflammatory cytokine rates in plasma and hippocampus, compared to mice without restriction [88]. Secondly, methionine restriction affects the oxidative stress balance in the liver by upregulating transsulfuration pathways of methionine, increasing H2S plasmatic levels [56, 93]. Indeed, the transsulfuration pathway after methionine catabolism leads to converting homocysteine to cysteine, producing H2S as a byproduct when degrading homocysteine or cysteine further [97]. This is interesting because H2S is a known liver metabolite improving lipid metabolism, increasing glucose production and decreasing oxidative stress in a physiological state [98]. Here, authors showed H2S production was correlated positively with protein anabolism and negatively with protein catabolism [56]. So methionine restriction alleviates oxidative stress via endogenous H2S production, improving liver metabolism and the inflammatory status in mice with DIO.

After acute exercise, methionine plasma concentrations decrease and plasmatic metabolites of transsulphuration and glutathione biosynthesis increase. In humans, one study looked at acute and long term exercise and plasma metabolite concentrations, including sulphur-containing metabolites [99]. Twenty six sedentary middle age men were recruited as normoglycemic men with a body mass index (BMI) below 27 kg/m^2^ or dysglycemic men with a BMI between 27 and 32 kg/m^2^ and impaired fasting plasma glucose, glucose tolerance or insulin resistance. Authors interspersed two acute bicycle challenges by 12 weeks of high intensity resistance and endurance exercise. After long term exercise, the major plasma metabolite concentrations changes were related to glutathione biosynthesis and transsulphuration. These changes correlated with change in insulin sensitivity, the strongest correlation rate being with total plasmatic cysteine. Taken together, these results suggest that exercise might decrease methionine and metabolites alongside diet and improve insulin resistance in overweight patients.

To recapitulate**,** methionine restriction has positive effects on health, as seen in rodent models of diet-induced obesity and a human study reviewed here. Moreover, one fails to normalize metabolic responses in DIO rat models when moderately restricting protein but keeping the methionine levels at a correct level [87]. However, adding another sulfur amino acid, cysteine, can reverse the effects seen above in mice models concerning energy expenditure, weight gain, lipid, insulin and adiponectin plasmatic levels [90, 100]. This is probably because cysteine is downhill of the methionine signaling pathway, so its addition bypasses the effects of methionine restriction [97]. Finally, in normal weight humans with a low cysteine and methionine diet, the glutathione biosynthesis pathway adapts: methionine plasmatic and urinary metabolites are low, but total cysteine is unchanged, suggesting compensatory mechanisms occur to counter low amino-acid availability [101]. Future studies are needed restricting all sulfur containing amino-acids in rodents and humans to validate this data and optimize therapeutic interventions.

### Other amino acids

#### Tryptophan as another potential treatment for obesity

As reviewed recently, tryptophan restriction in a diet promotes longevity in mammals [84, 86]. When moderately restricted, tryptophan increases thermogenesis and food intake, whereas it decreases energy expenditure, food intake, body weight, fat and lean mass when severely restricted [102]. Solon-Biet and colleagues fed C57BL/6 J non obese mice with the same rate of protein but varying proportions of BCAAs compared to the control diet [103]. Obesity developed in the groups with the highest proportions of BCAA and their food intake was the highest. Interestingly, tryptophan, threonine and methionine levels were stable across groups and supplementing the group with twice the normal amount of BCAA by tryptophan or threonine reversed hyperphagia to control levels. So, in a high BCAA diet an amino-acid imbalance in tryptophan and threonine occurs, with secondary hyperphagia [103]: this phenomenon rather than the toxic effect of BCAAs leads to subsequent obesity, hepatosteatosis and shorter lifespan.

**Fig. 2:**
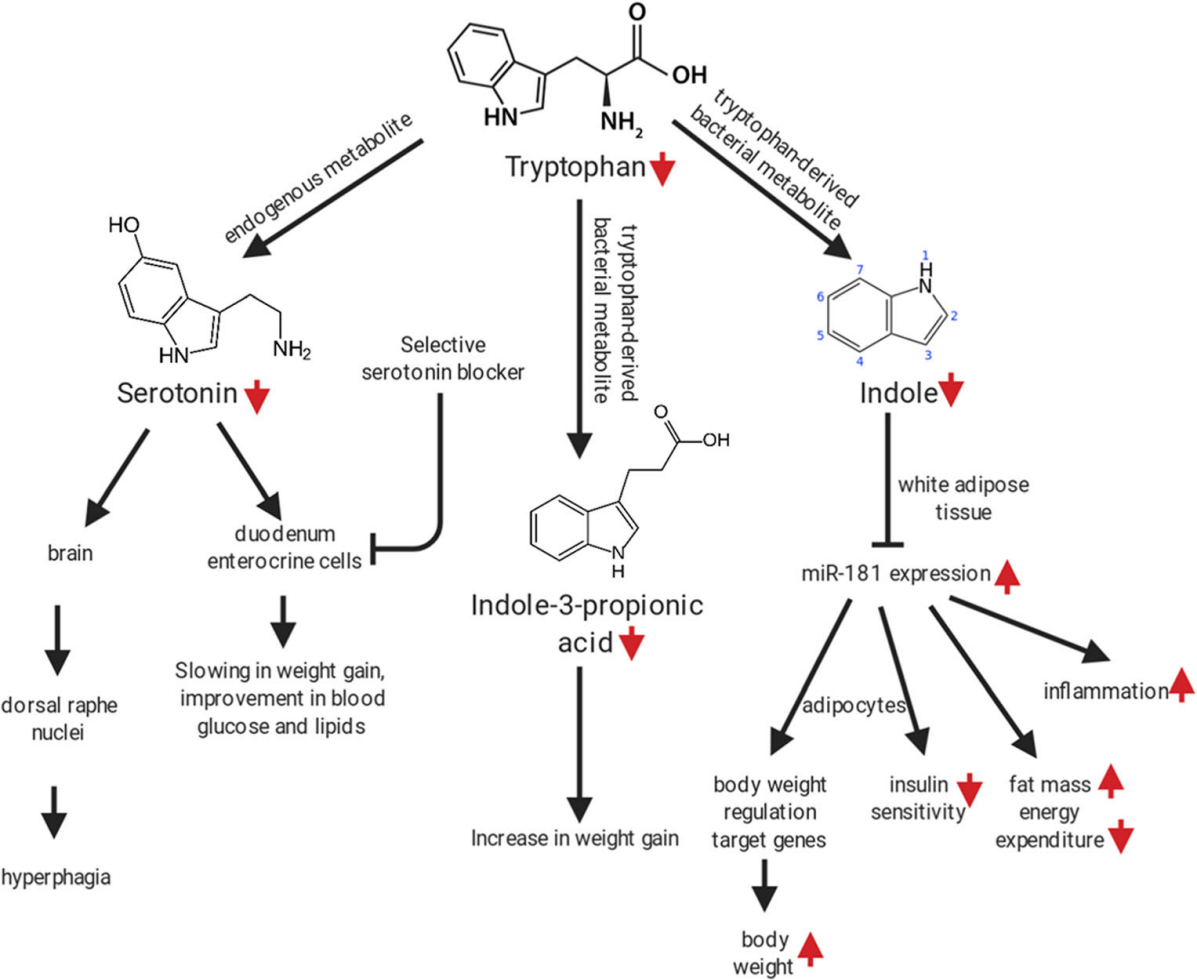
impact of tryptophan restriction or imbalance on weight gain, glucose tolerance, body composition, energy expenditure, macrophage polarization, food intake and behavior. Figure constructed using the Biorender software

Besides tryptophan itself, its metabolites (endogenous such as serotonin or derived from gut bacteria) play a role in the disruption of the metabolism and appetite observed in obesity (Fig. 2). Pan et al. treated male C57BL/6 DIO mice with a selective, intestinal serotonin blocker, decreasing serotonin serum and intestine levels. They found a slowing in weight gain and improved blood glucose and lipid values compared to controls [104]. Besides endogenous serotonin, treating rats fed a standard diet with antibiotics lowered plasmatic indole-3-propionic acid, a tryptophan gut bacteria derivative, and displayed higher weight gain in animals [105]. In addition, colonizing germ free mice with microbiota from controls or feeding a high fat diet, tryptophan-free diet to C57BL/6 mice, lowered indole, another tryptophan metabolite which plasma levels are low in obese adolescents [106]. Finally, tryptophan restriction effects vary depending on age and only partially simulate total amino acid restriction. In 6 week-old male DIO rats, tryptophan restriction affected body composition and hepatic protein sensing changes differently then total amino acid restriction [107].

If we summarize these studies, direct or indirect tryptophan restriction through other AA imbalance impacts both endogenous metabolites and bacterial-derived metabolites in contradictory ways depending on which metabolite is modified (Fig. 2). However, in non-obese adult rats, inducing a tryptophan-rich diet with 8.5 g/kg of tryptophan reduced body weight, food intake, increased tryptophan metabolites and impaired behavioral tests compared to inducing a control diet [105]. Moreover, restricting histidine, another aromatic amino acid impacted food intake, body weight and composition and liver protein synthesis [107]. So further research on total aromatic amino acid restriction must be planned in a similar way as for BCAAs and with different time points to take into account age specific effects.

#### Glutamate as another potential treatment for obesity

The role of glutamate is debated in obesity. Indeed, giving the sodium salt of glutamic acid, a food additive, at 2 mg/kg of body weight in rats induces central obesity, glucose intolerance, NAFLD, oxidative stress and inflammation [108]. These results were rarely reproduced in humans, probably because this amount is not physiological. In this review, we focus on the role of glutamate intake and concentrate on glutamate as a neuromodulator for appetite regulation and adiposity in obesity.

Glutamatergic signaling modulates appetite. In the hypothalamus of transgenic mice developed to reveal glutamatergic receptors in neurons by fluorescence, food withholding and subsequent leptin injection in mice reveals a novel population of glutamatergic signaling neurons, sensitive to leptin [109]. Orexigenic neuromodulators inhibit and anorexigenic neuromodulators excite these neurons in electrophysiological experiments on mice brain slices. Finally, inhibiting food intake by glutamatergic signaling seems to function via exciting anorectic POMC neurons. However, in DIO male C57BL/6 J fed a diet with 10%, 45% or 60% fat for 6 months, metabolite analyses show plasmatic and hippocampal levels of glutamine increase with 60% HFD, without glutamate change in the brain [110]. So glutamate as a metabolite might impact appetite regulation via hypothalamic glutamatergic neurons but also through its precursor, glutamine.

Besides its role as a neurotransmitter, glutamate also contributes to adiposity and body weight in mammals and humans. A study monitored the content in free amino acids of the milk of 65 healthy lactating women prospectively alongside their children’s body weight. They found glutamate more highly concentrated in the faster weight gain compared to the slow weight gain group [111]. In adult pigs, glutamate administration to a standard diet reduced body fat weight and increased colonic short chain fatty acids [112]. In a human subsample of a multicentric European cohort investigating risk factors associated with childhood obesity, authors studied food preference and anthropometrics in 1839 randomly selected children [113]. Preference for the umami flavor, induced by 1% monosodium glutamate addition to high fat crackers correlated with lower BMI z-score, lower arm circumference and lower fat mass. In a cohort of 59 healthy women with BMIs between 20 and 41.1 kg/m^2^, glutamate has been identified as a biomarker for visceral adipose tissue [114]. So glutamate at physiological doses is a potential therapeutic target for appetite regulation, cognitive improvement, body weight or adiposity control, and a biomarker for visceral adiposity.

## Conclusion

To conclude, varying proteins, both in quality and quantity can be considered as a treatment for the body composition, metabolic syndrome parameters and appetite regulation in obese patients, and these effects vary across age groups. Therapeutic interventions like bariatric surgery also affect protein intake, which can be unfavorable for the surgical outcome, and this could be avoided by a combination of protein supplementation and exercise. Finally, specific amino-acid restrictions such as BCAA, methionine, tryptophan and glutamate instead of protein modulation as a whole might be potential future therapeutic interventions, with a need for more human studies of validation.
